# Targeting novel regulated cell death: Ferroptosis, pyroptosis and necroptosis in anti‐PD‐1/PD‐L1 cancer immunotherapy

**DOI:** 10.1111/cpr.13644

**Published:** 2024-04-09

**Authors:** Li Yu, Ke Huang, Yixiang Liao, Lingzhi Wang, Gautam Sethi, Zhaowu Ma

**Affiliations:** ^1^ Health Science Center Yangtze University Jingzhou Hubei China; ^2^ Department of Urology Jingzhou Central Hospital, Jingzhou Hospital Affiliated to Yangtze University Jingzhou Hubei China; ^3^ Department of Pharmacology Yong Loo Lin School of Medicine, National University of Singapore Singapore Singapore; ^4^ Cancer Science Institute of Singapore, National University of Singapore Singapore Singapore; ^5^ NUS Centre for Cancer Research (N2CR), National University of Singapore Singapore Singapore

## Abstract

Chemotherapy, radiotherapy, and immunotherapy represent key tumour treatment strategies. Notably, immune checkpoint inhibitors (ICIs), particularly anti‐programmed cell death 1 (PD1) and anti‐programmed cell death ligand 1 (PD‐L1), have shown clinical efficacy in clinical tumour immunotherapy. However, the limited effectiveness of ICIs is evident due to many cancers exhibiting poor responses to this treatment. An emerging avenue involves triggering non‐apoptotic regulated cell death (RCD), a significant mechanism driving cancer cell death in diverse cancer treatments. Recent research demonstrates that combining RCD inducers with ICIs significantly enhances their antitumor efficacy across various cancer types. The use of anti‐PD‐1/PD‐L1 immunotherapy activates CD8^+^ T cells, prompting the initiation of novel RCD forms, such as ferroptosis, pyroptosis, and necroptosis. However, the functions and mechanisms of non‐apoptotic RCD in anti‐PD1/PD‐L1 therapy remain insufficiently explored. This review summarises the emerging roles of ferroptosis, pyroptosis, and necroptosis in anti‐PD1/PD‐L1 immunotherapy. It emphasises the synergy between nanomaterials and PD‐1/PD‐L1 inhibitors to induce non‐apoptotic RCD in different cancer types. Furthermore, targeting cell death signalling pathways in combination with anti‐PD1/PD‐L1 therapies holds promise as a prospective immunotherapy strategy for tumour treatment.

## Graphical abstract

This review summarises the novel roles of ferroptosis, pyroptosis, and necroptosis in anti‐PD‐1/PD‐L1 immunotherapy. Targeting non‐apoptotic regulated cell death in combination with anti‐PD1/PD‐L1 therapies holds promise as a prospective immunotherapy strategy for tumour treatment.

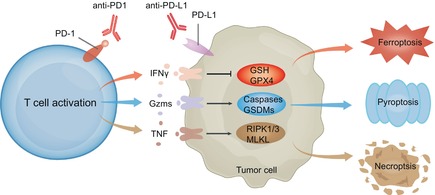

## 1 INTRODUCTION

The application of immune checkpoint inhibitors (ICIs) in cancer immunotherapy has shown remarkable success in clinical settings.^1^, ^2^ Since their initial FDA approval in 2011, ICIs have rapidly become integral to various cancer treatment protocols.^3^ This approval has revolutionised cancer care and paved the way for significant progress in ICIs and other immuno‐oncology therapies.^4^, ^5^ ICIs, including antibodies targeting programmed cell death 1 (PD1) and programmed cell death ligand 1 (PD‐L1), have demonstrated effectiveness against diverse cancers, such as melanoma, non‐small‐cell lung cancer, and renal cancer.^6^ Consequently, the development of anti‐PD‐1/PD‐L1 antibodies has garnered considerable attention in the field of cancer immunotherapy. However, only a subset of patients with cancer benefit from immune checkpoint blockade (ICB), suggesting alternative PD‐1/PD‐L1‐related pathways contributing to immunopathogenesis and treatment resistance.^7^ Currently, combining immunogenic cell death (ICD) in cancer cells with anti‐PD‐1/PD‐L1 treatment enhances immune therapy by activating the T cell‐based immune system and reinforcing the antitumor immune response, ultimately leading to effective tumour therapy in vivo.^8^

Cell death has been implicated in various disorders stemming from deregulated or dysfunctional cell death signals.^9^ It can be categorised into two groups: accidental cell death triggered by uncontrolled biological processes due to accidental injury stimuli and regulated cell death (RCD) governed by integrated signalling cascades and well‐defined mechanisms of action.^10^ Growing evidence indicates that specific RCD subroutines play crucial roles in carcinogenesis and could pave the way for several potential therapeutic strategies.^11^ Among the various described types of cell death, ferroptosis, pyroptosis, and necroptosis are the most comprehensively understood.^12^ Recent research underscores the involvement of these cell death forms in the development and progression of various diseases, including cancers.^13^ Different forms of RCD have been found to modify the tumour microenvironment (TME) by releasing pathogen‐ or damage‐associated molecular patterns (PAMPs or DAMPs), thereby enhancing the efficacy of cancer therapies.^14^ As cancers frequently display resistance to apoptosis, inducing non‐apoptotic RCD emerges as a promising strategy for cancer treatment.

Certain recent reviews have discussed the role of RCD in tumour immunity.^15^, ^16^, ^17^ However, the specific contributions of ferroptosis, pyroptosis, and necroptosis in anti‐PD1/PD‐L1 immunotherapy within the context of cancer remain largely unexplored. This review aims to outline the functions of ferroptosis, pyroptosis, and necroptosis in anti‐PD1/PD‐L1 immunotherapy when combined with nanomaterials and PD‐1/PD‐L1 inhibitors. Targeting cell death signalling pathways using anti‐PD1/PD‐L1 presents a promising combined strategy for cancer therapy.

## 2 ANTI‐PD1/PD‐L1 CANCER IMMUNOTHERAPY

Recently, ICI therapy has revolutionised the treatment landscape for various cancer types.^18^ ICIs, encompassing inhibitors targeting PD‐1, PD‐L1, and cytotoxic T‐lymphocyte antigen‐4 (CTLA‐4), have demonstrated unprecedented efficacy in treating numerous cancers.^19^ The CTLA‐4 and PD‐1/PD‐L1 axes play critical roles in maintaining immune homeostasis, suppressing inflammatory responses, and potentially facilitating immune evasion by cancer cells.^20^ Blocking these PD‐1/PD‐L1 and CTLA‐4/B7 axes has led to improved overall survival and increased response rates in diverse cancer types.^21^

The development and progression of tumours intricately involve immune cell infiltration, immune modulation, and immune evasion within the TME.^22^ Tumour immunity can promote tumour progression by modulating tumour cell characteristics, selecting resilient cancer cells within the microenvironment, and establishing a favourable TME.^23^ Immune cell functions are regulated by both co‐inhibitory and co‐stimulatory receptors.^24^ Notably, the introduction of T cell‐targeted ICIs, such as CTLA‐4 and PD‐1 or PD‐L1, represents a significant breakthrough in cancer treatment.^25^ The approval of ipilimumab in 2011 marked the first ICI targeting CTLA‐4.^26^ Subsequently, monoclonal antibodies targeting PD‐1 and PD‐L1 have been developed and widely used in anticancer therapies.^27^ Anti‐PD‐1/PD‐L1 antibodies constitute primary immunotherapy for various cancers, including melanoma, lung cancer, breast cancer, and renal cancer.

Immune checkpoints, like PD‐1, are inherent regulatory pathways in immune cells, monitoring and modulating immune activity.^28^ PD‐1, an inhibitory receptor expressed on activated B cells, T cells, regulatory T cells (Tregs), macrophages, and natural killer (NK) cells, interacts with its ligands PD‐L1 and PD‐L2 (B7 family) found on various cells, including B cells, T cells, dendritic cells (DCs) and macrophages.^29^, ^30^, ^31^ This binding inhibits T cell activity, suppresses proliferation, induces T cell tolerance, and promotes cell death.^32^ The interaction between PD‐1 on T cells and PD‐L1 on tumour cells constitutes a significant obstacle in the cancer‐immune cycle, leading to the apoptosis of T cells and the inhibition of T cell activation and proliferation.^33^ Inhibiting PD‐1/PD‐L1 signalling has transformed cancer therapy by releasing exhausted tumour‐responsive CD8^+^ T cells within the TME. Recent studies also highlight the superior therapeutic efficacy of tumour‐specific memory cells from draining lymph nodes against tumours upon transfer, exhibiting responsiveness to PD‐1/PD‐L1 blockade.^34^, ^35^

ICI immunotherapy has demonstrated remarkable efficacy across various cancer types.^36^ Nivolumab, the first PD‐1 monoclonal antibody, gained approval for melanoma treatment in 2014.^37^, ^38^ Combining nivolumab and ipilimumab, which target PD‐1 and CTLA‐4, respectively, significantly improved overall survival rates in patients with advanced melanoma in a phase 3 clinical trial.^39^ Similarly, monoclonal antibodies targeting PD‐L1, such as atezolizumab and durvalumab, have demonstrated efficacy in clinical trials across a range of cancers.^40^, ^41^, ^42^ However, PD‐1/PD‐L1 blockade therapy exhibits effectiveness in only a subset of patients with specific tumour types, including non‐small‐cell lung cancer, melanoma, bladder cancer, and kidney cancer.^43^, ^44^

## 3 REGULATORY CELL DEATH AND CANCER IMMUNOTHERAPY

**FIGURE 1:**
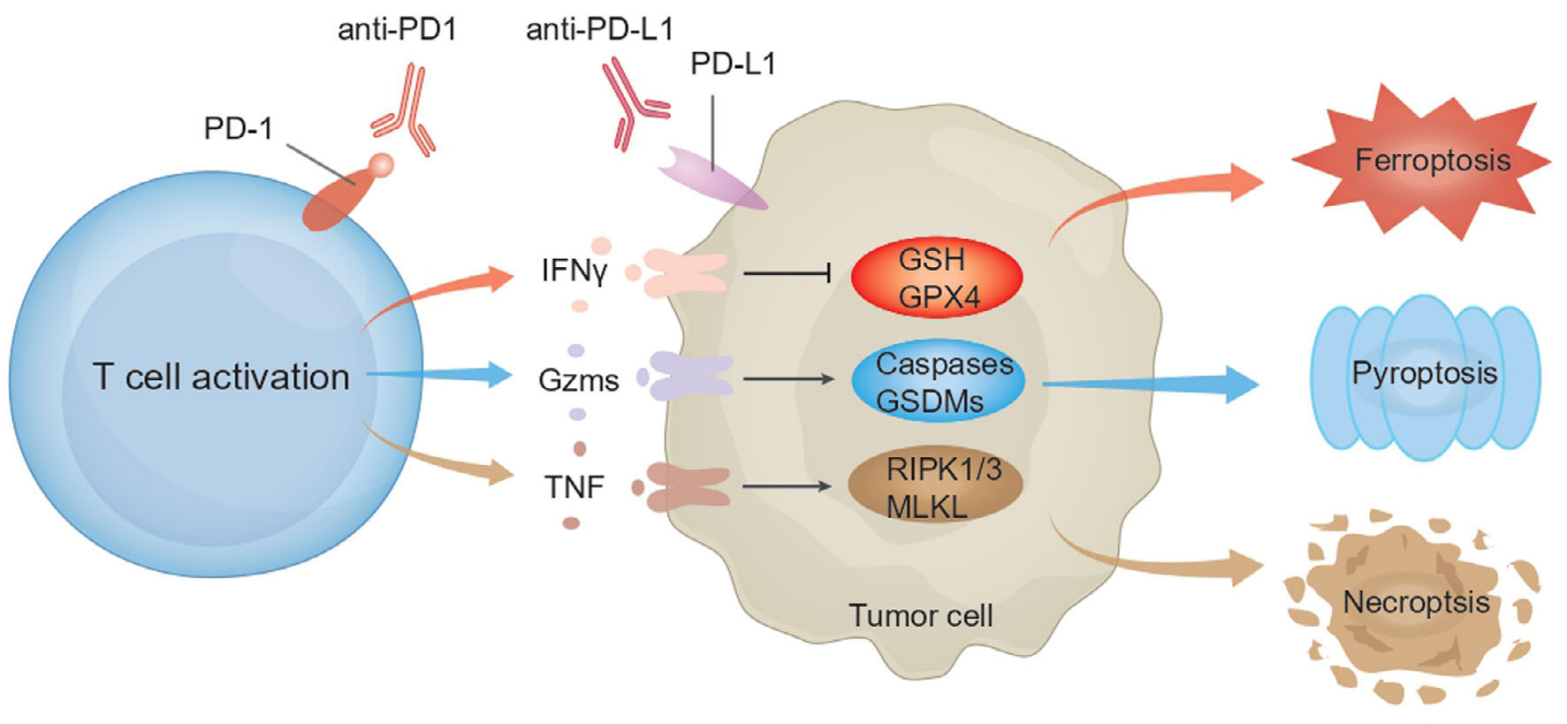
Anti‐PD‐1/PD‐L1 therapy induces non‐apoptotic regulated cell death (RCD) of tumour cells. Anti‐PD1/anti‐PD‐L1 therapy enhances T cell activation and promotes the release of IFNγ, Gzms, and TNF, which can trigger multiple cell death signalling pathways to induce non‐apoptotic RCD, such as ferroptosis, pyroptosis, and necroptosis.

Targeting the cell death pathway has emerged as a promising avenue to enhance the effectiveness of tumour immunotherapy.^16^, ^45^ Programmed cell death, or RCD, not only plays a crucial role in embryogenesis but also significantly impacts disease development, especially cancer. The ability of cancer cells to evade cell death is a hallmark of cancer itself.^46^ Recent studies suggest that combining non‐apoptotic forms of RCD with ICIs can synergistically enhance antitumor activity across various cancer types.^15^ Therefore, a comprehensive understanding of the regulatory mechanisms behind ferroptosis, pyroptosis, and necroptosis in the context of ICB is pivotal for advancing antitumor immunotherapy. In the following sections, we summarise the emerging roles of ferroptosis, pyroptosis, and necroptosis in anti‐PD1/PD‐L1 immunotherapy. Moreover, combination therapy with anti‐PD‐1/anti‐PD‐L1 antibodies not only amplifies T cell activation but also triggers the release of coherent signals such as interferon‐γ gamma (IFN‐γ), granzymes (Gzms) and TNF, capable of initiating multiple cell death signalling pathways, leading to non‐apoptotic forms of RCD, including ferroptosis, pyroptosis and necroptosis (Figure 1).

### 3.1 Ferroptosis in cancer immunotherapy

Ferroptosis represents an iron‐dependent form of RCD characterised by necrotic morphology.^47^ Its morphological features encompass abnormal mitochondria, reduced cristae, condensed membrane structures, and outer membrane rupture.^48^ As a new form of RCD, ferroptosis can either be activated or inhibited based on specific cellular conditions. The hallmark of ferroptosis lies in the accumulation of iron‐catalysed phospholipids containing polyunsaturated fatty acid in cell membranes, causing peroxidation at lethal levels.^49^, ^50^ This lipid peroxidation induces membrane rupture, elevates membrane permeability, and ultimately culminates in cell death.^51^ Glutathione peroxidase 4 (GPX4) stands as the primary detoxifying enzyme, mitigating lethal lipid peroxidation (LPO) by utilising glutathione (GSH) as a substrate. The system Xc^−^ antiporter, consisting of SLC7A11 and SLC3A2, plays a crucial role in importing extracellular cystine in exchange for intracellular glutamate.^52^, ^53^ Once inside the cells, cystine is rapidly reduced to cysteine, vital for GSH biosynthesis.^54^ Ferroptosis has been implicated in various diseases and functions as a mechanism for suppressing tumours. Inducing ferroptosis holds therapeutic promise for treating neoplastic diseases and other conditions.^55^, ^56^

Emerging evidence has revealed that ferroptosis exerts a critical role in regulating tumour immunity, particularly in PD1 checkpoint blockade therapies. Studies indicate that cytotoxic CD8^+^ T cells can increase LPO in tumour cells, inducing ferroptosis and enhancing the effectiveness of PD1 checkpoint blockade therapy.^57^ Additionally, IFN‐γ signalling triggered by ICB can synergistically induce and amplify tumour ferroptosis alongside arachidonic acid, resulting in effective tumour regression.^45^ Recent studies also reveal that inhibiting HnRNP L decreases PD‐L1 expression and enhances antitumor immunity by destabilising YY1 mRNA in castration‐resistant prostate cancer (CRPC), thereby promoting T cell‐mediated cancer cell ferroptosis.^58^ These findings shed light on the mechanisms underlying ferroptosis in anticancer immunotherapy, offering valuable insights for novel therapeutic strategies in cancer treatment.

### 3.2 Pyroptosis in cancer immunotherapy

Pyroptosis represents an emerging form of programmed necrosis characterised by plasma membrane lysis, cell swelling, chromatin fragmentation, and proinflammatory content release.^59^ Unlike apoptosis, pyroptotic cells undergo chromatin condensation and DNA fragmentation while retaining intact nuclei.^60^ In the canonical inflammasome pathway, pattern recognition receptors detect danger signals, initiating inflammasome activation and downstream pathways that activate caspase‐1, triggering pyroptosis, and the secretion of IL‐18 and IL‐1β.^61^, ^62^ In the noncanonical inflammasome pathway, human caspase‐11 (or caspase‐4/5 in mice) recognises and binds to microbial lipopolysaccharides, leading to its activation.^63^, ^64^ Activated caspase‐11 initiates a protease cascade inducing pyroptosis and also activates caspase‐1 by cleaving gasdermin D (GSDMD).^65^ Additionally, caspase‐3 activates gasdermin E (GSDME), inducing pyroptosis,^66^ while caspase‐8 cleaves gasdermin C (GSDMC), inducing pyroptosis and shifting TNFα‐induced apoptosis to pyroptosis.^67^ Pattern recognition receptors detecting PAMPs and DAMPs initiate large ASC supramolecular assemblies linking NLR to caspase‐1. This complex activates pyroptosis via caspase‐1 or murine caspase‐11 and human caspase‐4/5 activation.^60^ Pyroptosis and the inflammasome pathway have significant implications for inflammation, immunity, and various disease processes. For instance, lower GSDMD expression in gastric cancer cells compared to adjacent non‐cancer cells suppresses pyroptosis in tumour cells, fostering cancer cell proliferation.^68^ Another study revealed that triggering pyroptosis can rescue chemotherapy‐resistant pancreatic and lung cancer cells, thereby overcoming chemotherapy resistance in cancer.^69^

Pyroptosis has the potential to modulate the TIME by releasing proinflammatory cytokines, tumour‐associated antigens, and DAMPs. This, in turn, triggers intratumoral inflammatory responses, promotes the infiltration of tumour‐specific cytotoxic T cells, converts ‘cold’ tumours to ‘hot’ tumours, and ultimately enhances the efficacy of ICB therapy. Certain cancer treatments restore the immune surveillance against cancer by inducing pyroptosis. Combining pyroptosis‐inducing therapies with ICB therapy may yield synergistic effects in cancer treatment.^70^ For instance, recent studies indicate that inducing pyroptosis and subsequent inflammation robustly triggers antitumor immunity, synergising with anti‐PD1 therapy.^71^ In a glioblastoma model, the combination therapy of oncolytic virotherapy with anti‐PD‐1 antibody significantly improved efficiency by inducing pyroptosis in tumour cells.^72^

### 3.3 Necroptosis in cancer immunotherapy

Necroptosis, a form of regulated necrosis, has been extensively studied in various biological contexts, encompassing both homeostasis and cancer.^73^ In contrast to apoptosis, necroptotic cells exhibit distinct morphological features such as organelle swelling, increased cell volume, and loss of membrane integrity, eliciting an immune response.^74^ Key proteins involved in necroptosis mainly include receptor‐interacting protein kinase 1 (RIPK1), receptor‐interacting protein kinase 3 (RIPK3), and the phosphorylation of its substrate mixed lineage kinase domain‐like pseudokinase (MLKL). RIPK1 activity can be specifically inhibited by necrostatin‐1, the first well‐defined RIPK1 activity inhibitor.^75^ In the presence of inhibited caspase‐8, activated RIPK1 binds to downstream RIPK3 and phosphorylates RIPK3, leading to the formation of the ripoptosome. Subsequently, RIPK3 phosphorylates MLKL, forming the necrosome.^76^ Within the necrosome, MLKL, a recognised functional substrate of RIPK3, undergoes oligomerisation, culminating in necroptotic cell execution.^77^, ^78^

Necroptosis, similar to other programmed cell death mechanisms like ferroptosis and pyroptosis, has garnered recent attention for its substantial involvement in cancer cell initiation, proliferation, tumour necrosis, and the immune responses within tumours.^79^ RIPK3‐mediated necrosis has been shown to suppress myeloid leukaemia development specifically by mediating the necrosis of myeloid leukaemia cells.^80^ Additionally, methylation near the transcription start site can silence RIPK3 expression in tumour cells. Thus, treatment with hypomethylation drugs may potentially improve prognosis by restoring RIPK3 expression and enhancing sensitivity to chemotherapeutic drugs.^81^ While tumour cell necroptosis aids tumour clearance, it alone does not fully elucidate the entire antitumor effect of necroptosis inducers, indicating a link between antitumor immunity and necroptosis.^82^ Activation of RIPK1/RIPK3 in necroptotic cells promotes the activity of CD103^+^ cDC1‐ and CD8^+^ leukocytes, instigating antitumor immune responses. This immune response synergises with ICB, specifically targeting PD‐1 (β‐PD‐1), to enhance durable tumour clearance.^83^

## 4 REGULATORY CELL DEATH IN ANTI‐PD1/PD‐L1 THERAPY

Given the emergence of resistance mechanisms against apoptosis, tumour cells often exhibit deficiencies in executing cell death. Consequently, researchers increasingly focus on targeting non‐apoptotic routes of RCD to improve the efficiency of anticancer immunotherapy, especially in advanced ICB and nanobiotechnology contexts.^84^ In the subsequent sections, we provide an overview of RCD and its significant contributions to enhancing the synergistic effects of tumour anti‐PD1/PD‐L1 therapy (Table 1). Compared to single‐agent anti‐PD1/PD‐L1 therapy, inducing non‐apoptotic forms of RCD, such as ferroptosis, pyroptosis, or necroptosis, may substantially overcome resistance to anti‐PD1/PD‐L1 therapy, rendering cancer cells more susceptible to immunotherapy.

**TABLE 1:** Summary of published immune checkpoint inhibitors in regulated cell death (RCD)‐based cancer therapy.

Cancer type	Treatment modality	RCD inductions	Molecular axis	Functions	Immune features	Treatment strategy	References
Ferroptosis							
Melanoma	PFG MPNs	GW4869/Fe^3+^	SLC7A11/SLC3A2/GSH/GPX4/Fe^2+^/Fe^3+^, IFNγ signalling pathway /Fenton reaction	PTT with antiexosomal PD‐L1 enhanced ferroptosis and induced potent antitumor immunity	ICD	Enhances PD‐L1 checkpoint blockade	87
Melanoma	HGF NPs	GW4869/Fe^3+^	SLC7A11/SLC3A2/GSH/GPX4/Fe^2+^/Fe^3+^, IFNγ signalling pathway /Fenton reaction	Reverses immunosuppression by exosomal PD‐L1 is associated with increased ferroptosis	ICD	Enhances PD‐L1 checkpoint blockade	88
Melanoma	DOX‐TAF@FN	DOX/TAF	SLC7A11/GSH/GPX4/ROS, SystemXc^−^/Fenton reaction	Enhances ferroptosis and strengthens the antitumor immune response	ICD	Combined anti‐PD‐L1 therapy	89
Melanoma	DZ@TFM	Fe^3+^/Mn^2+^	SLC7A11/SLC3A2/GSH/GPX4/Fe^2+^/Fe^3+^, SystemXc^−^/Fenton reaction	Favours the ferroptosis‐immunotherapy cyclical synergism	ICD	Enhances PD‐L1 checkpoint blockade	90
Melanoma	Pa‐M/Ti‐NCs	PA/Ti	Fe^2+^/Fe^3+^, Fenton reaction	Promotion of ferroptosis/immunomodulation synergism in cancer	ICD	Enhances PD‐1 checkpoint blockade	91
Melanoma	RCH NPs	Hemin, Celecoxib, Roscovitine	GPX4, Fenton reaction, COX‐2/PGE2 pathway, Cdk5 pathway	Cascade enhances ferroptosis and disrupts the inflammation‐relate immunosuppression in tumour therapy	ICD	Enhances PD‐L1 checkpoint blockade	92
Melanoma	Targeting arachidonic acid metabolism	IFNγ	ACSL4, IFNγ signalling pathway	Mediates immunogenic tumour ferroptosis	ICD	Enhances PD‐L1 checkpoint blockade	45
Melanoma	Targeting CD36	Fatty acids	ROS, IFNγ signalling pathway	Inhibits ferroptosis in CD8^+^ T cells effectively, promotes antitumor activity, and possesses superior antitumor efficacy in combination with anti‐PD‐1 therapy	ICD	Combined anti‐PD‐1 therapy	93
Melanoma	Targeting CAMKK2	‐	GPX4/GSH, AMPK–NRF2 pathway	Increase responses to anti‐PD‐L1 therapy by enhancing ferroptosis	ICD	Combined anti‐PD‐1 therapy	94
Leukaemia	GNPIPP12MA	FTO inhibitor	GSH/GPX4, GSH‐mediated signalling pathways	Induces ferroptosis and enhances the response to PD‐L1 blockade	ICD	Enhances PD‐L1 checkpoint blockade	97
Leukaemia	GCMNPs	Ferumoxytol	GPX4/ROS, IFNγ signalling pathway/Fenton reaction	Induces ferroptosis and anti‐PD‐L1 synergistically to promote T cell immune response	ICD	Enhances PD‐1/PD‐L1 checkpoint blockade	98
Breast cancer	Fe3O4‐SA@PLT	SAS/Fe^2+^	GSH/GPX4, SystemXc^−^/Fenton reaction	The efficacy of PD‐1 checkpoint blockade therapy is significantly enhanced by the induced ferroptosis	ICD	Combined anti‐PD‐1 therapy	100
Breast cancer	Targeting TYRO3	‐	SLC3A2/GPX4, AKT/NRF2 pathway	Enhances ferroptosis and sensitises resistant tumours to anti‐PD‐1 therapy	‐	Combined anti‐PD‐1 therapy	101
Colorectal cancer	iRGD‐bcc‐USINP	Iron	GSH, Fenton reaction	Induces ICD and mediates tumour cell ferroptosis	ICD	Enhances PD‐L1 checkpoint blockade	103
Colorectal cancer	ZnP@DHA/PYRO‐Fe	Chol‐DHA/PYRO‐Fe	ROS, Fenton reaction	Induces ferroptosis and promotes anti‐PD‐L1 checkpoint blockade	ICD	Enhances PD‐L1 checkpoint blockade	104
Colorectal cancer	BEBT‐908	PI3K and HDAC	GPX4/SLC7A11, IFN‐γ‐STAT1 signalling pathway	Effectively induces immunogenic ferroptosis of tumour cells and potentiates cancer immunotherapy	ICD	Combined anti‐PD‐1 therapy	105
Lung cancer	ZVI‐NPs	GSK3/β‐TrCP	GPX4/SLC7A11, AMPK/mTOR signalling pathway	Synergistically induces ferroptosis in tumour cells and reprograms the immunosuppressive microenvironment	ICD	Enhances PD‐1/PD‐L1 checkpoint blockade	107
Malignant pleural effusion	RT‐MPS	RIBE	ROS, STAT1/MAPK signalling pathway	Enhances broad antitumor effects and immunogenic death mainly by ferroptosis	ICD	Combined anti‐PD‐1 therapy	109
Prostate cancer	Targeting HnRNP L	‐	SLC7A11/GPX4/SLC3A2, IFN‐γ‐STAT1 signalling pathway	Destabilises the stability of YY1 mRNA and enhances T cell‐mediated cancer cell destruction, reducing PD‐L1 expression and promoting antitumor immunity in CRPC	ICD	Enhances PD‐L1 checkpoint blockade, combined anti‐PD‐1 therapy	58
Hepatocellular carcinoma	Man@pSiNPs‐erastin	pSiNPs‐Cy7/erastin	SOCS3‐STAT6‐PPAR‐γ pathway	Targets macrophage ferroptosis and protumoral polarisation	ICD	Combined anti‐PD‐L1 therapy	108
Pyroptosis							
Prostate cancer	YBS‐BMS NPs‐RKC	YBS/BMS‐202	Caspase‐1/GSDMD	Synergising immunogenic pyroptosis induction and ICB, stimulating a powerful antitumor immunity	ICD	Enhances PD‐1/PD‐L1 checkpoint blockade	112
Glioblastoma	rAAV‐GSDMD^NT^ (rAAV‐P1)	GSDMD^NT^	GSDMD	Induces pyroptosis and prolongs survival in preclinical cancer models	‐	Combined anti‐PD‐L1 therapy	72
Breast cancer	NP–GSDMA	GSDMA3	GSDMA3	Pyroptosis‐induced inflammation triggers robust antitumor immunity	ICD	Combined anti‐PD‐1 therapy	71
Breast cancer	GOx‐Mn/HA	GOx	GSDMD	Regulating tumour glycometabolism induces pyroptosis and promotes PD‐L1 expression in tumour cells	‐	Combined anti‐PD‐L1 therapy	114
Breast cancer	Hypoxia conditions induce activation of the nPD‐L1–p‐STAT3 complex	PD‐L1/p‐Stat3	GSDMC/Caspase‐8, nPD‐L1–p‐STAT3 /caspase‐8‐mediated pathway	Converts apoptosis of cancer cells into pyroptosis to promote necrosis	‐	PD‐L1 acts as an upstream regulatory molecule	118
Melanoma/Colorectal cancer	Hyperactive dendritic cells	Inflammasome (IL‐1β, IFNγ, IL‐2, CTLs)	GSDMD	Leads to pyroptosis and stimulates durable antitumor immunity	ICD	Combined anti‐PD‐1 therapy	116
Pancreatic cancer	USP48‐GSDME	GSDME	GSDME	Promotes GSDME expression and enhances cell antitumor immune response, thereby exerting antitumor effects	‐	Combined anti‐PD‐1 therapy	115
Ovarian	Downregulates the rate‐limiting enzymes in the mevalonate pathway	HMGCR/HMGCS1	GSDMD	Synergises with anti‐PD‐L1 therapy by driving inflammasome‐regulated immunomodulating pyroptosis	‐	Combined anti‐PD‐L1 therapy	111
Necroptosis							
Melanoma	αHSP70p‐CM‐CaP	CM/αHSP70p/CpG	DAMP	Combined with anti‐PD‐1 therapy and promotes tumour regression by killing target cells in vivo	ICD	Combined anti‐PD‐1 therapy	122
Melanoma	An AAV vector encoding for a constitutively active form of RIPK3	Enzyme RIPK3	RIPK1/RIPK3 activation, NF‐κB signalling pathway	Induces tumour cell necroptosis and synergises with ICB to enhance durable tumour clearance	ICD	Combined anti‐PD‐1 therapy	83
Melanoma	A compound that can override the requirement for ADAR1 inhibition and activate ZBP1	ZBP1/RIPK3	RIPK3/MLKL	Induces ZBP1‐dependent necroptosis and converts ICB unresponsiveness	ICD	Combined anti‐PD‐1 therapy	123
Osteosarcoma	TNF‐α‐loaded nanoplatforms	TNF‐α	TNF‐α	Switches the tumour cell into an endogenous vaccine and promotes antitumor immunity of anti‐PD‐1/PD‐L1	ICD	Combined anti‐PD‐1/anti‐PD‐L1 therapy	125
Chronic infection	Improving antigen‐specific CD8^+^ T cell responses	Caspase‐8	Caspase‐8, RIPK3	Activation of necroptosis and increases cell death	ICD	Correlated positively with PD‐1 expression	126
Cholangiocarcinoma	The combination of a necroptosis‐based therapeutic approach with ICIs	DAMPs	MLKL, RIPK1‐RIPK3	Promotes PD‐L1 expression and has greater antitumor activity in combination with ICB	ICD	Correlated positively with PD‐L1 expression	127

### 4.1 Ferroptosis in anti‐PD1/PD‐L1 immunotherapy

#### 4.1.1 Ferroptosis in melanoma

##### Ferroptosis in melanoma and NPs, iron‐based nanoparticles

**FIGURE 2:**
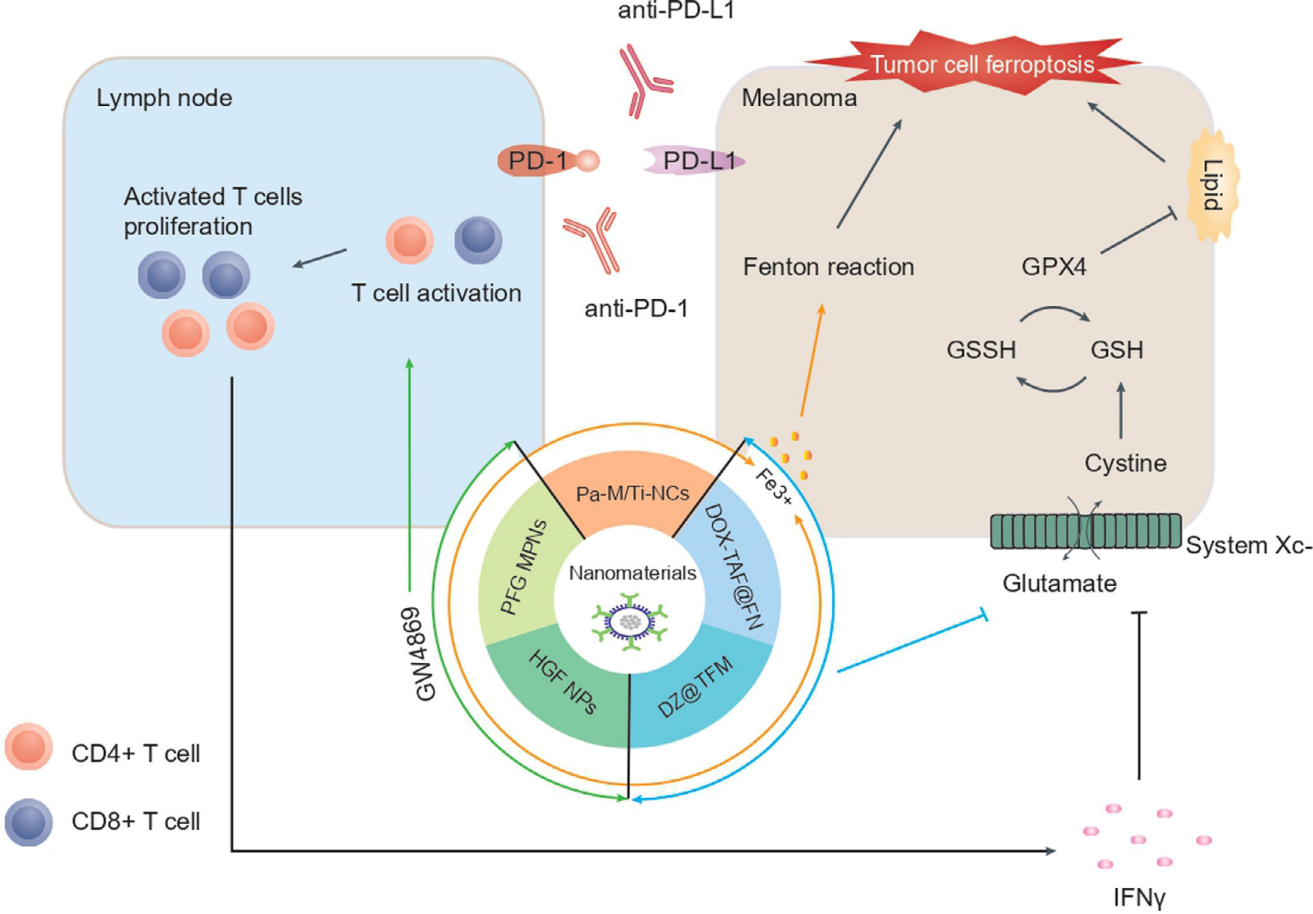
Combining anti‐PD1/anti‐PD‐L1 and nanomaterials in ferroptosis‐based cancer therapy. The combination of anti‐PD1/anti‐PD‐L1 and nanomaterials induces the ferroptosis of tumour cells through three pathways: **(**i**)** Enhancing the ferroptosis induced by promoting the Fenton reaction, with Fe ions released from nanomaterials. (ii) Nanomaterials (DZ@TFM and DOX‐TAF@FN) promote ferroptosis of tumour cells by inhibiting glutamate‐cystine antiporter system Xc^−^ and downregulating SLC7A11 and GPX4. **(**iii**)** GW4869 released from HGF NPs and PFG MPN significantly reduced the generation of tumour‐derived exosome, leading to the enhancement of an antitumor immune response and increasing the level of IFN‐γ cytokine released by T cells to inhibit system Xc^−^ and enhance the ferroptosis.

Melanoma, an invasive skin cancer originating from melanocytes in the skin,^85^ poses numerous challenges despite substantial improvements in suggested therapeutic tactics.^86^ In melanoma models, combining anti‐PD1/anti‐PD‐L1 with nanomaterials to induce ferroptosis exposes tumour antigens, enhancing tumour cell immunogenicity and reinforcing immunotherapy effectiveness. The combination induces ferroptosis through three pathways: activation of the immune system's antitumor response, inhibition of the system Xc^−^ and promotion of the Fenton response (Figure 2). Therefore, combining anti‐PD1/anti‐PD‐L1 with nanomaterials offers considerable advantages in ferroptosis‐based cancer treatment. Novel cancer therapies leveraging nanomaterials targeting ferroptosis combined with anti‐PD1/anti‐PD‐L1 are promising.

Targeting ferroptosis and combining anti‐PD1/anti‐PD‐L1 with nanomaterials encompasses various design concepts. First, nanoparticles releasing exosome inhibitors activate the antitumor response, inhibit the glutamate‐cystine antiporter system Xc^−^ and enhance ferroptosis. Notably, GW4869‐mediated PD‐L1‐based exosomes hinder T‐cell revitalisation and promote ferroptosis. Studies have designed phototheranostic metal‐phenolic networks (PFGMPNs) and HACA‐Fe nanoparticles (HGF NPs) encapsulating FIN (Fe^3+^) and GW4869 in semiconductor polymer assembly.^87^, ^88^ GW4869 diminishes exosomal PD‐L1 secretion, activating T cells, and boosting IFN‐γ production. Consequently, IFN‐γ inhibits ferroptosis‐related molecules‐ SLC7A11, SLC3A2, GSH, and GPX4‐ enhancing ferroptosis induced by released Fe^3+^ from HGF NPs and PFG MPNs. This fosters a sustained immune response to the tumour.^87^, ^88^

Second, nanomaterials induce ferroptosis in tumour cells by inhibiting the Xc^−^ system and downregulating key ferroptosis molecules. Xu et al. constructed DOX‐TAF@FN using a multifunctional nanoplatform loaded with DOX‐loaded tannic acid (TA)‐iron network for cancer chemo‐/chemodynamic therapy. DOX‐TAF complexes possess excellent biocompatibility and stable pH‐responsive release of both DOX and Fe. These complexes trigger cancer cell ICD via DOX chemotherapy and TAF‐induced Fe‐generated chemodynamic therapy, enhancing tumour cell ferroptosis. This is characterised by lipid peroxide accumulation, Xc^−^ system activation, and GPX4 downregulation.^89^ The released TA converts Fe^3+^ to Fe^2+^ and Fe^2+^ and reacts with tumour cell hydrogen peroxide via the Fenton reaction, producing hydroxyl radicals and GSH, enhancing ferroptosis and inducing antitumor immunity. The ICD cancer cells synergise with additional anti‐PD‐L1, upregulating immune cell expression at tumour sites and significantly downregulating Tregs, efficiently suppressing tumours.^89^ Other studies have shown that combining DNAzyme (DZ)‐mediated PD‐L1 inhibition enhances ferroptosis‐induced melanoma immunotherapy.^90^ For instance, a study constructed a metal‐phenolic networks (MPNs) nanoplatform to promote tumour antigen presentation and cyclical synergism of ferroptosis‐immunotherapy. They regulated melanoma immune pathways by assembling MPN‐loaded PD‐L1 inhibiting DZ through TA with Fe^3+^/Mn^2+^ metal‐ions complexation. Upon intracellular delivery, the complex released TA and converted Fe^3+^ to Fe^2+^, triggering the Fenton reaction to induce ferroptosis. Simultaneously, DZ activated by Mn^2+^ effectively silenced PD‐L1, further enhancing the antitumor immune response.^90^

Lastly, nanomaterial‐induced ferroptosis directly promotes the Fenton reaction. Besides various nanoparticles inducing the Fenton reaction, Zhang et al. discovered that combining iron (Fe), checkpoint antibodies and a TGF‐β inhibitor with engineered nanoparticles (NPs) synergistically enhances the antitumor immune response. This elevates hydrogen peroxide (H_2_O_2_) levels in M1 macrophages, initiating a Fenton reaction that produces hydroxide ions (OH^−^) and ensuing ferroptosis of cancer cells, along with releasing tumour antigens, thereby increasing TIME's immunogenicity.^91^ Furthermore, a study designed a self‐amplifying nanodrug (RCH NPs) using human serum albumin to co‐assemble celecoxib (an anti‐inflammatory drug), roscovitine (a cyclin‐dependent kinase inhibitor) and hemin (ferric porphyrin). Within the RCH NPs, hemin catalyses the conversion of endogenous H_2_O_2_ into cytotoxic hydroxyl radicals (•OH) via the Fenton reaction, leading to LPO and thereby inducing ferroptosis in tumour cells. Celecoxib disrupts inflammation‐related immune suppression, while roscovitine blocks the Cdk5 pathway, suppressing IFN‐γ‐ induced PD‐L1 transcription, eliminating acquired immune resistance, and enhancing the antitumor effect.^92^ Therefore, the synergistic effect of ferroptosis and immune regulation by promoting the Fenton reaction offers promise for effective tumour treatments.

##### Ferroptosis in melanoma and NNPs, non‐iron‐based nanoparticles

In the context of melanoma, the combination of anti‐PD1/anti‐PD‐L1 with non‐nanomaterials can also induce ferroptosis, enhancing tumour cell immunogenicity and improving immunotherapy efficacy. For instance, a recent report demonstrated that arachidonic acid combined with PD1/anti‐PD‐L1 blockades triggers tumour ferroptosis via the IRF1/ACSL4 axis, presenting a potential anticancer therapeutic strategy.^45^ Additionally, studies revealed that blocking CD36 or inhibiting ferroptosis in CD8^+^ T cells effectively restores their antitumor functions. Combining CD36 deletion with anti‐PD‐1 antibodies enhances cancer immunotherapy.^93^ Furthermore, Wang et al. identified Calcium/calmodulin‐dependent protein kinase Quiescing 2 (CAMKK2) negatively regulating ferroptosis in melanoma via the AMP‐activated protein kinases‐NRF2 pathway. They also showcased that CAMKK2 suppression enhances the effects of ferroptosis inducers and anti‐PD‐1 therapy effects in preclinical melanoma models.^94^ These findings unveil new avenues for combined therapies involving ferroptosis induction and immune checkpoint drugs.

#### 4.1.2 Ferroptosis in leukaemia

**FIGURE 3:**
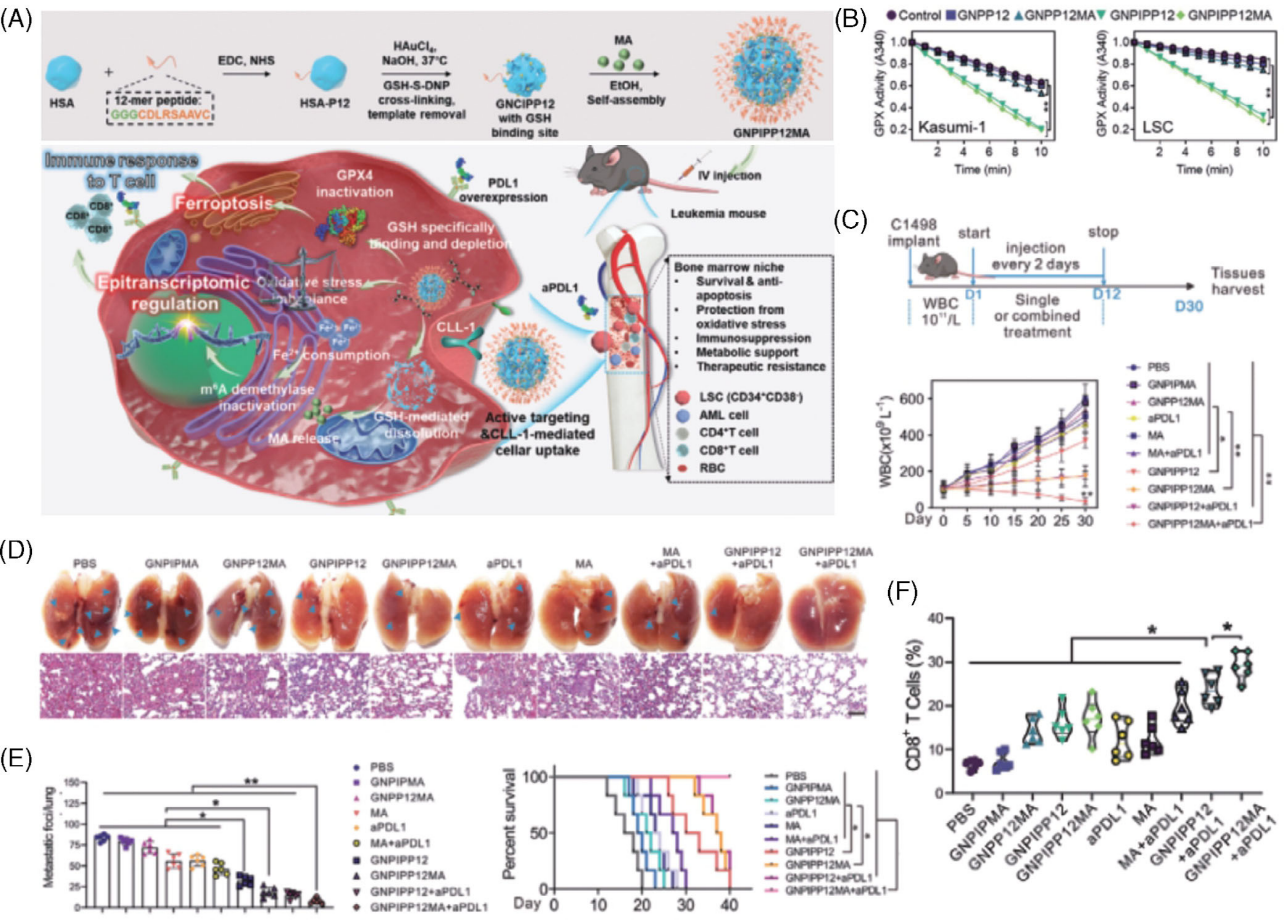
GNPIPP12MA‐induced ferroptosis cooperated with an anti‐PDL1 antibody to reduce AML growth in vivo. (A) Preparation of glutathione‐imprinted nanocomposites loading FTO inhibitor (GNPIPP12MA) and Mechanism of GNPIPP12MA inhibit leukaemia stem cell via targeting N6‐methyladenosine RNA methylation for enhanced anti‐leukaemia immunity. (B) GPX4 activity in Kasumi‐1 and LSC cells treated with 50 μg mL^−1^ of indicated nanoparticles. (C) Therapeutic regimen of C1498 leukaemia model and WBC counts for indicated groups (n = 6). (D) Representative images of spleen lung with H&E‐stained sections in the indicated groups. Scale bar = 10 μm. (E) Metastatic lesion area of the lung in the indicated groups and the 40‐day survival curve of mice in the indicated groups. (F) Flow cytometry assay of CD8^+^ T cell populations in the indicated groups. Data are mean ± SD; *p <0.05, **p <0.01. *n* = 6/group. aPDL1: anti‐PDL1. [Adopted from Cao et al.^97^]

Studies utilising murine leukaemia models evaluated the efficacy of ICI combined with ferroptosis inducers. Investigations explored combining anti‐PD‐1/anti‐PD‐L1 therapies with nanomaterials in the context of ferroptosis, introducing novel treatments for acute myeloid leukaemia (AML), a range of heterogeneous myeloid malignancies.^95^ Despite therapeutic drug administration being a core strategy for AML, its effectiveness is often hindered by low bioavailability, toxic side effects, and the need for intravenous administration.^96^ For instance, Cao et al. developed GSH‐bioimprinted nanocomposites loaded with an FTO inhibitor, GNPIPP12MA, to synergistically inhibit FTO and deplete GSH. GNPIPP12MA targeted AML cells and leukaemia cells in the bone marrow niche, inducing ferroptosis by reducing intracellular GSH levels. Moreover, GNPIPP12MA increased overall m6A modification in leukaemic stem cells, enhancing the efficacy of the PD‐L1 blockade by promoting cytotoxic CD8^+^ T cell infiltration (Figure 3).^97^ Similarly, Li et al. leukocyte membranes coated with poly (lactic‐co‐glycolic acid) encapsulating glycyrrhetinic acid (GCMNPs) to promote tumour targeting, tumour‐homing ability, and in vivo toxicity reduction. GCMNPs induce ferroptosis in AML and colorectal cancer (CRC) cells by downregulating Gpx4, leading to increased LPO levels. In vivo, the combination of GCMNPs and ferumoxytol enhanced PD‐1/PD‐L1 blockade, activating T cells against leukaemia and colorectal tumours.^98^

#### 4.1.3 Ferroptosis in breast cancer

The combination of ferroptosis‐based cancer therapy and immunotherapy in breast cancer has emerged as a novel nanomedicine design strategy.^99^ Studies indicated that loading sulfasalazine (SAS) into magnetic nanoparticles (Fe_3_O_4_) and masking them with a platelet membrane (Fe_3_O_4_‐SAS@PLT) enhanced tumour treatment outcomes. Mechanistically, SAS inhibits cysteine uptake, suppressing tumour growth and inducing ferroptosis. In the presence of SAS, Fe^2+^ released from Fe_3_O_4_ activates the Fenton reaction, generating excessive •OH and inducing ferroptosis in cancer cells. Consequently, the collaboration between Fe_3_O_4_ and SAS inhibits the Xc^−^ pathway, triggering ferroptosis. Additionally, Fe_3_O_4_ nanoparticles synergistically induced ferroptosis and stimulated an antitumor immune response, promoting M1 polarisation of macrophages and effectively enhancing PD‐1 blockade therapy.^100^ Moreover, another study highlighted TYRO3 receptor tyrosine kinase inhibitors' efficacy in overcoming immunotherapy drug resistance in breast cancer. TYRO3 inhibition led to tumour cell ferroptosis through the AKT‐NRF2 pathway, altering the macrophage ratio and potentially overcoming resistance to anti‐PD‐1 therapy resistance. Therefore, the combination of Tyro3 inhibitors with anti‐PD‐1 drugs has the potential to overcome tumour cell drug resistance by promoting ferroptosis.^101^

#### 4.1.4 Ferroptosis in colorectal cancer

The combination of target anti‐PD‐1/anti‐PD‐L1 therapy with ferroptosis‐based cancer therapy offers multiple opportunities for CRC treatment.^102^ For example, Liang et al. designed ultrasmall single‐crystal Fe nanoparticles (bcc‐USINPs), which consisted of a zero‐valent Fe (0) core and an oxide shell (Fe_3_O_4_). Upon reaching the TME, the exposure of the Fe (0) core triggered the Fenton reaction, resulting in tumour suppression and inducing ferroptosis in various tumour cell lines. Importantly, bcc‐USINPs induced ICD, facilitating DC maturation, and electing an adaptive T cell response. Coupled with anti‐PD‐L1 antibodies, iRGD‐bcc‐USINPs mediated ferroptosis, enhancing the antitumor immune response and promoting immune memory.^103^ Moreover, ZnP@DHA/Pyro‐Fe, loaded with pyropheophorbide‐iron (Pyro‐Fe) and cholesterol derivative of dihydroartemisinin (DHA) induced ferroptosis in CRC, sensitising non‐immunogenic CRC and enhancing the therapeutic effect of anti‐PD‐L1 therapy.^104^ Additionally, the PI3K and HDAC dual inhibitor BEBT‐908 induced ferroptosis, activated the intracellular IFN‐γ‐STAT1 pathway, delayed cancer cell growth, and promoted cell ferroptosis. Combined with anti‐PD‐1 antibodies, BEBT‐908 improved immunotherapy efficacy and generated antitumor immune memory.^105^ These findings highlight the potential of combining anti‐PD‐1/anti‐PD‐L1 therapy with ferroptosis inducers for CRC treatment, presenting a promising clinical strategy.

#### 4.1.5 Ferroptosis in other diseases

The combination of ferroptosis‐based cancer therapy with anti‐PD1/anti‐PD‐L1 antibodies has shown enhanced antitumor efficacy in various cancers.^106^ For instance, zero‐valent‐iron nanoparticles (ZVI‐NP) displayed promise against lung cancer. In preclinical models, ZVI‐NP induced ferroptosis in lung cancer cells by disrupting mitochondria function, elevating oxidative stress and LPO. Remarkably, ZVI‐NP enhanced antitumor immunity by converting pro‐tumour M2 macrophages into antitumor M1 macrophages, reducing regulatory Tregs, and downregulating CTLA‐4 and PD‐1 in CD8^+^ T cells, thereby maximising antitumor effects.^107^ Similarly, a functional nanocarrier, Man@pSiNPs‐erastin, targeted xCT‐mediated macrophage ferroptosis and pro‐tumoural polarisation in a mouse hepatocellular carcinoma (HCC) model. Combining this treatment with anti‐PD‐L1 exhibited significant antitumor efficacy by targeting ferroptosis activation in TAMs.^108^ In a murine of malignant pleural effusion, irradiated tumour cell‐released microparticles (RT‐MPs) promoted tumour cell ferroptosis, triggered ICD, and improved tumour cell clearance via tumour‐associated macrophage polarisation combination treatment with RT‐MPs and PD‐1 antibody.^109^ In prostate cancer, heterogeneous nuclear ribonucleoprotein L (HnRNPL) inhibition reduced PD‐L1 expression, increased IFN‐γ production, and induced ferroptosis in CRPC cells through the STAT1/SLC7A11/GPX4 signalling pathways axis. Furthermore, HnRNPL knockdown combined with anti‐PD‐1 therapy showed enhanced CD8^+^ T cell‐mediated ferroptosis in CRPC.^58^

### 4.2 Pyroptosis in anti‐PD‐1/PD‐L1 immunotherapy

Pyroptosis is currently being investigated as a novel antitumor treatment strategy to promote antitumor immunity and overcome apoptosis resistance.^110^ Combining ICIs with pyroptosis induction presents a promising direction. In this section, we summarise the recent advances in combination therapy with anti‐PD1/anti‐PD‐L1 and other therapies to induce pyroptosis (Table 1).

Pyroptosis‐inducing cancer drugs synergise with anti‐PD1/anti‐PD‐L1 antibodies to stimulate a potent antitumor immune response and inhibit tumour progression. Simvastatin, when combined with ICB, promotes inflammasome‐regulated immunomodulatory pyroptosis in the ARID1A‐inactivated ovarian clear cell carcinoma mouse model.^111^ Wang et al. developed a pH‐responsive nano‐photosensitizer (YBS‐BMS NPs‐RKC) that combines immune checkpoint inhibition and immunogenic pyroptosis induction. Under near‐infrared light, the dual‐type photosensitised agent YBS produces multiple reactive oxygen species (ROS) on the cancer cell membrane, effectively triggering caspase‐1/GSDMD pathway‐induced immunogenic pyroptosis. This combination enhances tumour CD8^+^ T cell infiltration, cytotoxic secretion, and immune response while also effectively blocking immune evasion mediated by increased IFN‐γ secretion and PD‐L1 upregulation in tumour cells.^112^

Pyroptosis‐induced inflammation stimulates antitumor immunity when coupled with nanoparticles in combination with anti‐PD1/anti‐PD‐L1 therapy. For example, a biorthogonal system applying NP‐GSDMA3, which involves conjugating nanoparticles, phenylalanine trifluoroborate (Phe‐BF3), and the cancer imaging probe, allows investigation of the correlation between pyroptosis and immunity.^71^ When combined with nanoparticle‐mediated delivery, desilylation catalysed by Phe‐BF3 selectively cleaves target proteins, resulting in the controlled release of bioactive gasdermin A3 protein and inducing the pyroptosis of breast cancer cells in vivo.^113^ Similarly, combining pyroptosis‐inducing approaches with PD‐L1 treatments has been demonstrated to effectively repress tumour growth compared to single treatments by enhancing cancer immunity.^71^ Moreover, another study reported a novel strategy for packaging recombinant adeno‐associated virus (rAAV) expressing the N‐terminal gasdermin domain (GSDM^NT^) into tumour cells and inducing pyroptosis in preclinical cancer models, including glioblastoma. Simultaneously, rAAV recruits tumour‐infiltrating lymphocytes across the blood–brain barrier into the brain and enhances antitumor effects when used in combination with anti‐PD‐L1 drugs.^72^ Similarly, dual‐enzymatic nanoparticles were constructed via the hybridisation of nanozymes and glucose oxidase (GOx‐Mn/HA), which have the ability to induce cancer cell pyroptosis and promote PD‐L1 expression in tumour cells. This combination of nanoparticles with anti‐PD‐L1 also has a significant immunological memory effect, thus inhibiting tumour growth.^114^

Various factors induce pyroptosis by altering GSDMs and enhancing antitumor immunity.^115^, ^116^ Particularly, PD‐L1 can regulate pyroptosis, leading to tumour necrosis.^117^ Clinical trials have demonstrated that antibiotic chemotherapeutics enhance GSDMC‐mediated pyroptosis under hypoxia by combining STAT3 and PD‐L1.^118^ Targeting these regulators of pyroptosis may further advance their application in cancer therapy. Combination treatments with ICIs and other therapies hold promise for improving therapeutic effects in cancer immunotherapy.

### 4.3 Necroptosis in anti‐PD1/PD‐L1 immunotherapy

Necroptosis, an alternative mode of programmed cell death, circumvents apoptosis resistance and has shown the potential to stimulate and enhance antitumor immunity in cancer therapy.^119^ Emerging evidence has highlighted a strong association between antitumor immunity and necroptosis.^120^ In this section, we summarise recent advances in combining anti‐PD1/anti‐PD‐L1 with other therapies to induce necroptosis (Table 1).

As a potent inducer of ICD, necroptosis synergises with anti‐PD‐1/PD‐L1 antibodies, providing added therapeutic benefits in cancer treatment.^121^ For instance, a study developed a biomimetic artificial necroptotic cancer cell (α‐HSP70p‐CM‐CaP) vaccine containing HSP70 peptide, cancer membrane proteins (CM), and adjuvant CpG. This vaccine's delivery of immunogenic antigen materials and CpG to lymph nodes enhances DC maturation and antigen presentation, resulting in robust cellular immunity. αHSP70p‐CM‐CaP vaccination, when combined with anti‐PD‐1 antibodies in mouse melanoma models, led to target cell elimination and inhibited cancer progression in vitro.^122^ Additionally, delivering the RIPK3 gene to tumour cells using an AAV vector induced necroptosis in melanoma cells. Elevated RIPK1/RIPK3 expression in necroptotic cells activated the NF‐κB‐dependent signals pathway, promoting CD8^+^ leukocyte‐mediated immune responses, and in combination with anti‐PD‐1 therapy, improved durable tumour clearance.^83^ Moreover, CBL0137 triggered an antiviral response by inducing necroptosis in ICB therapy‐resistant melanomas. This small molecule‐induced Z‐DNA turnover activated ZBP1, culminating in RIPK3‐mediated necroptosis and immune system engagement against the tumour.^123^ Another study elegantly demonstrates that necroptosis‐driven inflammation through DCs works in combination with anti‐PD‐1 antibodies to inhibit the proliferation of melanoma.^124^ Similarly, in osteosarcoma, TNF‐α‐loaded liposomes induced ICD in tumour cells, leading to TNF‐α‐triggered necrosis, tumour‐specific antigen release, enhanced DC activation, and T cell infiltration when combined with anti‐PD‐1/PD‐L1 therapy.^125^

Various stresses regulate PD‐L1 expression, promoting cancer progression and impacting patient survival rates.^126^, ^127^ Targeting PD‐L1 and activating necroptosis could enhance their application in cancer immunotherapy. Combining necroptosis‐based therapy with ICIs may offer more effective treatment options for patients with cancer.

## 5 CONCLUSIONS AND PERSPECTIVES

The application of ICIs is limited in many cancers, with only approximately one‐third of patients showing a positive response.^128^ Converting these immune “cold” tumours into responsive ‘hot’ tumours remains a challenge. Targeting non‐apoptotic cell death and combining it with anti‐PD‐1/anti‐PD‐L1 therapy presents a promising breakthrough for overcoming this limitation and improving the efficacy of immunotherapy in the treatment of cancer. This review summarises the emerging roles of ferroptosis, pyroptosis, and necroptosis in anti‐PD‐1/PD‐L1 immunotherapy, emphasising the induction of non‐apoptotic RCD through nanomaterials and PD‐1/PD‐L1 inhibitors in various cancers. These strategies activate T cells, trigger cytokine release, and initiate cell death signalling cascades, potentially enhancing the efficacy of immunotherapy.

However, despite the progress in this domain, resistance to ICI treatment and treatment‐related toxicities continue to limit their clinical application.^36^ Several key questions warrant further exploration. First, while the anti‐PD‐1/PD‐L1 treatments activate T cells, the roles of other immune cells, such as DCs or NK cells, in promoting tumour regression and improving treatment effectiveness need deeper investigation.^129^, ^130^, ^131^ Second, exploring the combined use of other ICIs to induce RCD in cancer immunotherapy remains a challenge. Two of the most representative immune checkpoint pathways, CTLA‐4/B7 and PD‐1/PD‐L1, negatively regulate the immune function of T cells at various phases of T cell activation.^18^ Furthermore, the involvement of novel immune checkpoint molecules like V‐domain Ig suppressor of T cell activation and ectonucleotidases (CD39, CD73, and CD38) shows promise and requires extensive study for clinical benefits.^132^ Third, combining other antitumor strategies with anti‐PD‐1/PD‐L1 therapy could be a polytherapy strategy to enhance cancer cell death. Recently, five Phase III studies have demonstrated positive results, wherein PD‐1/PD‐L1 antibodies combined with antiangiogenic therapy induce vascular normalisation and antitumor immunity,^133^, ^134^ suggesting a potential triple therapeutic strategy in the future.

Several studies and clinical trials on anticancer immunity and non‐apoptotic cell death in cancers are currently ongoing (Table 2). Clinical trials are enrolling patients to explore non‐apoptotic cell death as both prevention and treatment for various cancers (e.g., NCT05493800, NCT04739618). Additionally, targeting regulators of RCD holds promise in enhancing the effectiveness and safety of immunotherapy. For instance, ongoing clinical trials are investigating the impact of talimogene laherparepvec on PD‐L1 expression in non‐muscle invasive bladder cancer through pyroptosis (NCT03430687). For the latest information, refer to the Clinicaltrials.gov database.

**TABLE 2:** Examples of clinical trials exploring the application of anticancer immunity and non‐apoptotic cell death in cancers.

ClinicalTrials.gov identifier	Study title	RCD	Cancer type	Intervention/treatment	Estimated enrollment	Primary purpose
NCT05493800	Evaluate the efficacy and safety for oral mucositis prevention of MIT‐001 in auto HSCT (Capella)	Ferroptosis	Haematologic Cancer	Drug: MIT‐001 Drug: normal saline	75 participants	Prevention
NCT03430687	Talimogene laherparepvec in treating patients with non‐muscle invasive bladder transitional cell carcinoma	Pyroptosis	Bladder transitional Cell carcinoma	Other: Laboratory biomarker analysis Biological: Talimogene laherparepvec	0 participants	Treatment
NCT04229992	Calcium: Magnesium balance, microbiota, and necroptosis and inflammation	Necroptosis	Colorectal Cancer	Dietary Supplement: Magnesium glycinate Dietary Supplement: Placebo	240 participants	Prevention
NCT02965703	Aspirin in preventing colorectal cancer in patients with colorectal adenoma	Necroptosis	Colorectal adenoma	Drug: Aspirin Procedure: Biospecimen collection Other: Placebo administration Other: Questionnaire administration Procedure: Rectal Biopsy	90 participants	Prevention
NCT04739618	Metastatic solid cancer clinical trial	Necroptosis	Metastatic cancer	Drug: Keytruda injectable product Drug: Yervoy injectable product Drug: GM‐CSF Procedure: Non‐ablative cryosurgical freezing	32 participants	Treatment
NCT03803774	Birinapant and intensity modulated re‐irradiation therapy in treating patients with locally recurrent head and neck squamous cell carcinoma	Necroptosis	Squamous cell Carcinoma	Drug: Birinapant Radiation: Intensity modulated radiation therapy	34 participants	Treatment

The future of cancer treatment lies in combining anti‐PD‐1/PD‐L1 immunotherapy with RCD mediators. Encouraging active participation in clinical trials for combination therapies is crucial for assessing their efficacy and safety, providing evidence for further in‐depth studies, and ultimately benefiting a larger population of patients with cancer.^15^ Both basic and clinical research are essential to deepen our understanding of the underlying pathophysiology of disease exacerbation during ICI therapy and identify predictive factors for immune‐related toxicity and antitumoral response.^135^ This knowledge will aid in selecting patients most likely to benefit from ICI treatment.

## AUTHOR CONTRIBUTIONS

Z.M. conceived the review article. L.Y. and K.H. contributed to the initial drafting of the manuscript, table preparation, visualisation, and overall editing. Y.L. helped to modify the manuscript. Z.M., G.S., and L.W. contributed to conceptualisation, funding, overall supervision, and supported review development, overall editing, and critical overall manuscript revision. All authors read and approved the final manuscript.

## CONFLICT OF INTEREST STATEMENT

The authors declare no conflict of interest.

## Data Availability

The data sharing is not applicable to this article.
